# Quality of life assessment in preterm children: physicians’ knowledge, attitude, belief, practice - a KABP study

**DOI:** 10.1186/1471-2431-13-58

**Published:** 2013-04-19

**Authors:** Marie-Ange Einaudi, Catherine Gire, Anderson Loundou, Pierre Le Coz, Pascal Auquier

**Affiliations:** 1 Timone University Hospital, Espace Ethique Méditerranéen; Aix-Marseille University, UMR 7268 Anthropologie bio-culturelle, Droit, Éthique & Santé (ADÉS), Marseille, France; 2 North University Hospital, Department of Neonatology, Marseille, France; 3 Aix-Marseille University, EA 3279 Self Perceived Health Assessment Research Unit, Marseille, France

**Keywords:** Decision-making, Ethical reflection, Prematurity, Quality of life assessment

## Abstract

**Background:**

The sequelae of extremely preterm birth have an impact on the quality of life (QoL) of these children. Standardized assessment of their QoL is rarely done in France. The aim of this study is to examine among all the types of physicians involved in the management of children born extremely preterm, their knowledge, use in routine practice and expectations concerning QoL assessment of these children using standardized questionnaires.

**Methods:**

Prospective survey among heads of obstetric, neonatal medicine and paediatric neurology departments, by means of questionnaires. Two qualitative methods were used: focus groups and Delphi method.

**Results:**

Seventy-eight physicians participated (obstetricians 24%, neonatologists 58%, paediatric neurologists 18%). The physicians considered QoL a relevant concept which they assessed subjectively. They expressed a need for information on methods of assessment. An ideal QoL questionnaire was described. Expectations regarding availability of QoL data were expressed from a medical, family and societal perspective. The impact of QoL measurement on the ethical aspect of decision-making was approached, in particular the potential impact of this tool on the decision made. Expectations were found to differ between specialties.

**Conclusion:**

This original study reports the perspective of experts on taking into consideration the QoL of children born extremely preterm. This is a subjective notion that is difficult to implement and which may influence therapeutic choices.

## Background

The short, medium and long-term outcome of extremely preterm children born before 28 weeks of amenorrhea (EP Children) is marked by the development of severe complications that can affect the quality of life (QoL) of the children and their families [1]. Quality of life is a recent concept proposed as a criterion for evaluating care strategies by supporters of these approaches [2,3] and recommended by major bodies such as the Haute Autorité de Santé in France, the European Medicines Agency and the Food and Drug Administration in the United States [4-6]. However, its use remains limited both in collective and in individual approaches. Few published works have investigated the reasons for this limited use [7]. While some conclusions of these works can be transposed depending on the specialties concerned, others are closely linked to the specific nature of the field of disease under consideration [8-10]. In paediatrics, the clinical benefit of standardized QoL assessment, particularly in chronic disease, has been demonstrated in a study in 303 paediatricians by Baars [11]. Nevertheless, only 17% of them used QoL questionnaires. The obstacles identified were lack of time for assessment, lack of an appropriate instrument and insufficient knowledge of the subject.

Can these results be applied to the specific field of neonatal medicine and to EP Children in particular? In this field, no data exploring these obstacles have been reported, with the exception of a qualitative study in focus groups [12], carried out among 14 professionals, which identified the main reasons for the absence of widespread application of QoL assessment: questions as to the concepts underlying measurement, insufficient knowledge of the appropriate tools available, fear that assessment may be stigmatized because it appears to be normative, questions as to the legitimacy of such evaluation. Although the study identified the principal obstacles, it did not provide information on the distribution of opinions on these topics among the professionals consulted.

The aim of this study was to determine, among physicians involved in the care of EP Children in France, their knowledge, use in routine practice and expectations concerning QoL assessment of children born preterm using standardized questionnaires, and the specific opinions of each specialty.

## Methods

The heads of all departments of obstetrics, neonatal medicine and paediatric neurology involved in the management of EP Children in France were invited to take part in the study (n = 161). Depending on the size of the department, the heads of department were asked to pass on the survey questionnaire to one to three physicians working under them in the department. So, the minimal number of expected answers was 161.

The questionnaire (see Additional file 1), developed after a phase of preliminary interviews [12], comprised around thirty closed questions and some open-ended questions on three main topics: knowledge of the concept of QoL and of existing assessment tools, use in actual practice (regularity of use, obstacles to use); expectations regarding the ideal tool; and expected impact of these approaches.

The questionnaire was sent out by e-mail and by post twice at an interval of 6 months, according to the Delphi method of achieving consensus [13]. (The Delphi method is a structured method which relies on a panel of experts, for gathering data within their domain of expertise. Experts answer questionnaires in two (as in our study) or more rounds. They are encouraged to revise their earlier answers in light of the replies of other members of their panel. The Delphi process aims to achieve a convergence of opinion on a specific issue.) In the second round, each responding physician was asked to confirm or modify their initial response in view of the synthesis of opinions of the first round of the survey. They were asked to answer 12 supplementary questions on the impact that QoL measurement could have on the ethics of decision-making.

Informed consent to participate in the study was obtained by each participant. Anonymity and confidentiality were maintained during the survey. Ethical approval was obtained from the University Hospital ethics committee of Marseille, France, and from French representative legal authority: the National Committee of Computing and Liberties (reference number 1427029).

### Statistical analysis

Qualitative variables are given as numbers and percentages, and quantitative variables as means and standard deviations. Comparisons according to medical specialty were made using the chi-square test or analysis of variance depending on the type of variable. Non-parametric tests were applied for variables with non-normal distribution. The threshold of significance of the tests was fixed at 5%. Statistical analyses were carried out using PASW Statistics (version 17.0) software.

## Results

The data presented below are the result of the consensus expressed after the second round of questions.

### Sociodemographic characteristics of the responding physicians (Table 1)

**Table 1:** Sociodemographic characteristics of the physicians

**Population**	**1st round of questioning**	**2nd round of questioning**
	n (%)*	n (%)*
Male gender	48 (61.5)	36 (61.0)
Experience		
Senior	72 (92.3)	56 (95.0)
Junior	6 (7.7)	3 (5.0)
Specialties		
Obstetrics	19 (24.4)	10 (17.0)
Neonatal medicine	45 (57.7)	36 (61.0)
Paediatric neurology	14 (17.9)	13 (22.0)
Type of practice with EP Children		
Perinatal period only	19 (24.4)	10 (16.9)
Overall management**	34 (43.6)	27 (45.8)
Other	25 (32.0)	22 (37.3)
Practice of long-term follow-up **	25 (32.0)	22 (37.3)
Experience with EP Children		
≤ 10 years	24 (30.8)	19 (32.2)
> 10 years	54 (69.2)	40 (67.8)

* The% is expressed as the percentage of respondents to the question.

** Overall management is defined as perinatal and short-term management (during hospitalization) and long-term management (follow-up beyond the age of 4 years).

EP Children, extremely preterm children.

Among the 161 minimal targeted physicians, 78 took part in the first round. Their mean age was 47.2 years (±9.9). They had been working with EP Children for an average of 17.3 years (±9.4). The participation rate in the second round was 75.6% (n = 59), and was significantly higher among paediatric neurologists (93%) and neonatologists (80%) than among obstetricians (53%). Only 12 physicians (20%) changed their opinion on at least one question compared with the first round. Sixty-three departments responded, a participation rate of 39%, and a mean of 1.2 physicians from each department completed the questionnaire.

### Knowledge and practice (Table 2)

**Table 2:** Knowledge and level of practice of the physicians

	n (%)*	**Obstetricians**	**Neonatologists**	**Paediatric neurologists**	***p***
		n 1(%)*	n 2(%)*	n 3(%)*	
Do you make use of the concept of QoL?	53 (70.7)	13 (68.4)	31 (73.8)	9 (64.3)	NS**
Do you read work on the subject?	44 (57.9)	7 (36.8)	28 (65.1)	9 (64.3)	NS
Do you know any questionnaires on QoL?	33 (42.3)	6 (31.6)	19 (44.2)	8 (57.1)	NS
Give some examples of questionnaires	31 (40.3)	6 (31.6)	17 (38.6)	8 (57.1)	NS
Do you assess your patients’ QoL in daily practice ?	43 (55.1)	9 (47.3)	24 (54.5)	10 (71.4)	NS
What proportion of patients do you assess ?					
•0-25%	14 (35.0)	6 (66.7)	6(27.3)	2 (22.2)	NS
•25-50%	6 (15.0)	1(11.1)	3(13.6)	2 (22.2)	
•50-75%	7 (17.5)	2(22.2)	5(22.7)	0 (0.0)	
•>75%	13 (32.5)	0 (0.0)	8 (36.4)	5 (55.6)	
For assessment, do you use:					
•Standardized questionnaire ?	2 (4.9)	0 (0.0)	0 (0.0)	2 (20.0)	NS
•Subjective assessment ?	33 (80.5)	7 (87.5)	18 (78.3)	8 (80.0)	
•Other means of assessment ?	6 (14.6)	1 (12.5)	5 (21.7)	0 (0.0)	
In your opinion, is assessment difficult to carry out because of lack of time ?	44 (62.0)	12 (75.0)	25 (59.5)	7 (53.8)	NS
In your opinion, should assessment be entrusted to other professionals ?	25 (35.6)	7 (43.8)	14 (34.1)	4 (28.6)	NS

*% of responders to the question.

** NS non significant.

QoL, quality of life.

QoL was identified as a relevant concept by all physicians, although in the supplementary questionnaire on the impact of QoL assessment on the ethics of decision-making, 36% declared that they found this notion “perplexing”. Seventy-two physicians (92%) gave a definition of QoL, which they saw as a multidimensional concept that mainly covers social well-being, psychological well-being and physical well-being.

Objective appreciation of QoL appeared theoretically possible for 95% (n = 56) of physicians who responded to the supplementary questionnaire, while in the first part of the survey less than 5% (n = 2) stated that they assessed the QoL of their patients in daily practice using standardized questionnaires.

No significant difference was found according to specialty. On the other hand, like the use of standardized questionnaires, the ability to name the instruments available was related to involvement in long-term follow-up (58% vs 35%, p = 0.05). While QoL was assessed in routine practice significantly more often by younger than by older physicians (100% vs 51%, p = 0.004), the younger physicians all assessed QoL subjectively (100% vs 42%, p = 0.03). All physicians who replied to the supplementary questionnaire (n = 59, 100%) stated that they were not sufficiently informed about these approaches.

The main obstacles to QoL assessment, suggested in the open-ended responses, were on the one hand the difficulty of measuring a subjective concept (12.8%, n = 10) and on the other hand, the difficulty of interpreting the results of assessment (7.7%, n = 6), which they considered needed systematic comparison with a control population. Lastly, time pressures were often cited, particularly by obstetricians (75%).

### Expectations regarding the ideal assessment instrument (Tables 3, 4)

**Table 3:** Desirable characteristics of a QoL assessment tool

	n (%)*
In your opinion, the maximum number of questions in a questionnaire should be between:	
• 5-10	11 (15.1)
• 10-15	39 (53.4)
• 16-20	20 (27.4)
• >20	3 (4.1)
In your opinion, the most appropriate type of response would be : *(several answers possible)*	
• A visual analogue scale	36 (42.9)
• Yes/no answers	26 (31.0)
• Multiple answers	15 (17.8)
• Open answers	7 (8.3)
In your opinion, the maximum time needed to reply to the questionnaire should be:	
• 5 minutes	6 (8.1)
• 10 minutes	43 (58.1)
• 15 minutes	19 (25.7)
• > 15 minutes	6 (8.1)
In your opinion, the questionnaire should be:	
• Personalized (choice of domains according to each child)	45 (60.8)
• Adapted to the child’s impairment fonction du handicap de l’enfant	31 (42.4)
• Identical for all EP Children in order to allow comparison between impairments	60 (82.2)
In your opinion, the questionnaire should yield:	
• A score for each of the dimensions explored	60 (79.0)
• A global QoL score	66 (86.9)
Do you think that the domains to be assessed should be the same whatever the age of the child between 6 and 10 years ?	50 (70.4)

*% of responders to the question.

QoL, quality of life.

EP Children, extremely preterm children.

**Table 4:** Ranking of QoL domains and modes of evaluation

	**Mean ranking ± SD**	**Rank**
Ranking of QoL domains, from the most important (1) to the least important (12)		
• Psychological well-being	2.3 (1.8)	1
• Physical well-being	2.7 (2)	2
• Family relationships	5.1 (2.6)	3
• Self-image	5.2 (3.4)	4
• Absence of impairment	5.4 (3.6)	5
• Cognitive function	5.7 (2.5)	6
• Physical symptoms	6.1 (3.2)	7
• Emotions and moods	6.4 (3.3)	8
• Social relationships	6.8 (3.2)	9
• General behavior	6.9 (2.7)	10
• Physical activities	7.7 (3)	11
• School life	7.8 (3)	12
Ranking of modes of evaluation, from the most preferable (1) to least preferable (5)		
• The child completes the questionnaire alone	2.5 (1.5)	1
• A parent answers for the child	2.6 (1.2)	2
• The parents help the child to answer the questionnaire	2.7 (1.2)	3
• The referring physician questions the child during the consultation	3.1 (1.7)	4
• A care team member not usually involved in the child’s care helps him/her to answer the questionnaire	4.1 (1.3)	5

SD, standard deviation.

QoL, quality of life.

The physicians defined the ideal instrument as a short questionnaire (10 to 15 questions), taking a maximum of 10 minutes to complete, based on visual analogue scales and yielding an overall score. For all physicians (100%), the three domains to be taken into account in priority to assess QoL were “psychological well-being”, “physical well-being” and “family relationships”. The two domains “psychological well-being” and “physical well-being” were ranked top in order of importance while “family relationships” and “self-image” were ranked third and fourth, respectively. Over 90% of physicians considered that the impact of prematurity on “social life” and on “self-image” should be taken into account in assessing QoL. As ideal responder to questions on QoL, the professionals clearly identified the child himself or herself, or the parents in place of the child or helping them to fill in the questionnaire.

### Expected impact (Table 5)

**Table 5:** Expected impact of availability of QoL data

	n (%)*	**Obstetricians**	**Neonatologists**	**Paediatric neurologists**	***p***
		n 1(%)*	n 2(%)*		
				n 3(%)*	
**Impact on society**					
• Provide information on the children’s outcome	72 (93.5)	19 (100)	43 (97.7)	10 (71.4)	*0.004*
• Make EP Children concern of society	64 (83.1)	16 (84.2)	36 (81.8)	12 (85.7)	NS
**Impact on the family**					
• Change the way parents see their child’s outcome	59 (76.7)	14 (73.7)	34 (77.3)	11 (78.6)	NS
• Improve support and help to the family	71 (92.2)	18 (94.7)	39 (88.6)	14 (100)	NS
**Medical impact**					
• Provide overall knowledge of the outcome of EP Children	77 (100)	19 (100)	44 (100)	14 (100)	NS
• Enhance the professional’s intuitive assessment	54 (75.0)	9 (56.3)	34 (81.0)	11 (78.6)	NS
• Integrate the concept of QoL in care practices	66 (86.9)	16 (88.9)	36 (81.8)	14 (100)	NS
• Give back the patient the feeling that he/she is central to the physician’s preoccupations	48 (65.7)	8 (50.0)	31 (72,1)	9 (64.3)	NS
• Improve communication between the care team, the child and the family	66 (85.8)	18 (94.7)	34 (77.3)	14 (100)	NS
• Give parents more precise information on the outcome of their child	64 (83.2)	19 (100)	34 (77.3)	11 (78.6)	*0.046*
• Rekindle ethical debate on neonatal resuscitation practices	68 (88.3)	17 (89.5)	39 (88.6)	12 (85.8)	NS
• Makes no contribution to my practice	5 (6.6)	0 (0.0)	5 (11.4)	0 (0.0)	NS

*% of responders to the question.

QoL, quality of life.

EP Children, extremely preterm children.

Opinion on the expected impact of the availability of QoL data did not significantly differ between specialties, with the exception of paediatric neurologists, of whom only 71% thought that QoL assessment would lead to improved information on the outcome of EP Children. More than 64% of physicians considered that assessment could have an impact on medical practice.

Analysis of open-ended responses revealed the nature of physicians’ expectations in various fields:

social, with the aim of implementing measures appropriate to the needs expressed, of weighing the consequences of current medical practices, and of redefining priorities in management,

family, aiming to take better account of the impact on those close to the child, siblings and parents,

moral, by giving new impetus to the ethical debate on respect for life and the approach of death.

### Decision-making (Table 6)

**Table 6:** Impact of QoL on decision-making

	n (%)*	**Obstetricians**	**Neonatologists**	**Paediatric neurologists**	***p***
		n 1(%)*	n 2(%)*	n 3(%)*	
If the data of the literature showed very poor QoL in EP Children, this information:					
- would affect your choices	50 (84.7)	9 (90.0)	31 (86.1)	10 (76.9)	NS
- would need to be relativized	38 (64.4)	4 (40.0)	27 (75.0)	7 (53.6)	NS
- should be given to parents as part of their information	48 (81.4)	10 (100)	28 (77.8)	10 (76.9)	NS
In your opinion, would therapeutic abstention be conceivable if future QoL was affected	51 (89.5)	10 (100)	32 (94.1)	9 (69.2)	NS

*% of responders to the question.

QoL, quality of life.

EP Children, extremely preterm children.

The ethical principles underlying QoL assessment, approached in the supplementary questionnaire, were approved by the majority of physicians: to show beneficence and humanity (81%, n = 48), to examine the child’s possibility of autonomy in the future (93%, n = 55), and as an expression of responsibility (91%, n = 52). The answers did not differ significantly according to specialty. However, the question of the child’s ability to autonomy was significantly more important to physicians with a perinatal activity than to those who followed the children in the long term (100% vs 82%, p = 0.01). While more than 88% of physicians (n = 68) considered that the availability of data on QoL would give new impetus to the ethical debate on neonatal resuscitation practices (Table 5, medical impact), nearly 90% (n = 51) of physicians who responded to the supplementary questionnaire would consider therapeutic abstention if QoL was compromised. Information on QoL was considered more particularly useful in the neonatal period by 64 physicians (83.1%) and in medium and long-term practice by 70 physicians (94.6%).

## Discussion

This study reveals both the value of QoL assessment in EP Children and the limited extent to which it is used, and explains the reasons. It highlights the physicians’ perspective by revealing different strategies and different concerns among those questioned. It completes the findings of Baars on the perspective of general paediatricians [11].

The limited participation in the survey may be explained by the length of the questionnaire and by the two rounds of questions. However, other reasons may be suggested. Quality of life could appear to come into conflict with personal convictions in the highly sensitive field of management of EP Children. Some physicians justified their declining to take part by the complexity of the subject and their lack of knowledge of it. Physicians’ lack of interest compared with nursing staff or social workers has already been observed [7]. The main limit of this observational study is based on the high number of studied variables, which does not allow to justify the number of subjects to be studied. However, the participation rate is comparable to that reported in similar surveys [11,14].

Interest in the subject, knowledge and practice varied according to age and specialty. Younger physicians gave more importance to QoL and appeared to put it into practice, as has already been described in a study comparing the perspectives of students and older physicians [15]. Similarly, the longer a child is followed by a professional, the better the latter can judge how the impairment is experienced and the impact it has. However, measurement of QoL is still little used and was reported by only two paediatric neurologists, and was certainly related to their experience of long-term follow-up. While the majority of physicians appeared interested in the topic, many had an indirect approach to QoL, as in the survey of Baars, where 61% of paediatricians assessed QoL informally [11]. The explanation given was lack of knowledge of the instruments available and the feeling that intuition is more appropriate than standardized measures, as has been reported in other disciplines [7,9].

Even if they do not use a standardized approach, the physicians believe that the domains to be evaluated are those that are classically described in chronic diseases [11]. They consider the consequences of the symptoms, notably neurodevelopmental problems and the impact on those close to the child [1,16-19]. The question of the autonomy appears to be an essential criterion, as described by Tanaka, whatever the age of the physicians questioned [15]. As in other disorders where the patient’s perspective is the most important, the physicians take into account the child’s perception and also that of the parents [1,20]. The factors that finally govern the QoL of extremely preterm children appear to be close to those classically described in paediatrics [11,21].

Physicians thus do not measure QoL but give responses that are socially “expected” of them: it is impossible for them to say that they are not interested in the patient’s QoL, the best viewpoint is that of the patient… that is, they give responses that are in contradiction with their practical experience. It is unlikely that this is related to total disinterest in the subject, but rather indicates following a conventional line of thought. The giving of expected responses is a mechanism identified in opinion surveys, and described as “selective reporting” [22].

Lastly, and this completes the study of Baars et al. [11], physicians describe an “ideal questionnaire” that would be likely to produce a QoL assessment that is relevant to their daily practice. This could direct the development of new tools, adapted to the population concerned. Obtaining the opinion of physicians on the best means of evaluating their patients’ QoL, and finding the most appropriate format of questionnaire, could encourage the practice of assessment [7,11].

The main reasons for not assessing QoL in routine practice, reported in the results, concur with those described in experts in other disciplines: principally a doubt as to the possibility of measuring subjective data, the impossibility of relating statistical data to an individual situation, the importance of not assimilating QoL and state of health, and more particularly QoL and impairment, the feeling of the lack of a reliable instrument, and lack of time [7-9]. Beyond these reasons, the physicians questioned added the fear that the population evaluated would be stigmatized.

Three principal expectations with regard to standardized QoL assessment were expressed.

Firstly, assessment of patients’ QoL gives better understanding of their needs, and so of the measures that need to be implemented to respond to them, as has already been documented by Greenhalgh [7]. Data on QoL would shed light on public decisions through better appreciation of the social and economic impact of extremely preterm birth. Moreover, the idea of “burden” appears in the literature [16,18,23] in relation to the heavy demands of medical and rehabilitation management, the inadequacy of support and assistance, the occupational impact and the impact on siblings. This raises questions on the intensive measures that are implemented in the perinatal period and are then reduced, both in human terms (limited number of places in medical and social facilities, recent and limited development of follow-up networks to support and assist the children and their families, difficulties in the provision of schooling) and in economic terms (non-reimbursement of some rehabilitation treatments, low benefits).

Secondly, QoL data may be used as a management instrument in the relationship with the patient. They can improve the care relationship and information on outcome, as already described by Stahlmann in relation to prematurity [17] or by Greenhalgh from a more general standpoint [7]. This information transmitted in the perinatal period is marked by incertitude as to the prognosis. The parents need more concrete information than the conventional data on morbidity, described as a percentage of sequelae or of mortality. The parents may wish to know more about the children’s feelings and experience, as has been described in other settings [19,24]. While information on the QoL of extremely preterm children appears to be useful to the parents, it is also useful to the physicians. Just as in chronic diseases where the patients’ daily life is affected, in extreme prematurity it is important to improve mutual knowledge between providers and recipients of care [21,25]. This is what seems to be sought by the physicians questioned in our study, and was already suggested by the paediatricians in the survey of Baars [11] and by Barlesi in a survey among physicians in a thoracic oncology network [9].

While the above two types of expectation are classically described in the literature, the third expectation that became apparent in the results is more original. Given the specific nature of the problem raised by extreme prematurity, in particular the decisional dilemma of the limits of viability [23], the experts could wish for a tool to rationalize individual decision-making, notably to reflect on the “burden-benefit” balance of neonatal management. A very large majority of the physicians questioned considered that such data could rekindle the ethical debate on neonatal resuscitation practices. The potential decisional impact of such information was mentioned by the majority of physicians questioned, notably the possibility of therapeutic abstention, which appears innovative in France in research on the subject. In a survey among 318 neonatologists in Pacific Rim countries, Martinez studied the factors that influenced neonatal resuscitation decisions at the limits of viability. Perception of poor quality of life, at the same level as the presence of severe congenital deformities, the parents’ wish not to resuscitate their child, or a high probability of neonatal mortality, was identified as a decisional factor by over 70% of the neonatologists questioned. It was not stated, however, whether consideration of QoL had an objective or subjective basis [26]. But in our survey this opinion was not shared by those who had expressed reservations as to the standardized use of QoL. While QoL is used as a theoretical notion to argue decisions on limitation or cessation of treatments, by projecting the patient’s future, it is never based on objective results, as has already been described [12]. One of the principal obstacles to the use of QoL as a decision-making tool is that it may be seen as a judgement, given the perinatal context, on lives that are or are not worth living, and so become assimilated to a tool of sacrifice. However, although these results are debatable (interpretation of the results, differences in methodology), nothing in the literature on extreme prematurity truly describes the QoL of lives that would not be worth living [1,27].

Quality of life assessment should be understood as an engagement to act. In the field of extreme prematurity, where “the child’s best interest” is one of the foundations of management [27,28], the finality of action cannot be limited to a short-term result, reflected in a mortality rate or a survival rate, without being tempered by more qualititative elements.

Quality of life may be approached from an ethical viewpoint if concern with QoL means caring for the patient attentively and benevolently, improving their well-being by responding to their needs, and developing means to alleviate dependence. In the “child’s best interest”, physicians must question themselves as to the appropriateness of their acts and the outcome of their patients [29]. Addressing the QoL of extremely preterm children thus appears as a question not only of deontology but also of professional responsibility, with the need to integrate QoL data into care, clinical and economic strategies, an issue which has already been raised in other disciplines [30,31].

## Conclusion

This study reports the perspective of experts on QoL assessment of children born extremely preterm. While all the physicians questioned were interested in the subject, the relationship with the patient and the expectations with regard to such assessment differed according to predefined categories, in particular mode of practice and age. The fairly consensual opinion was that QoL appeared as a subjective notion difficult to implement, but one that could however influence treatment choices. In another respect, it was shown that physicians’ opinion needs to be taken into account for the development of tools that can be used in their practice. Lastly, this study raises ethical questions, principally the conflict of values between “sacredness of life” and “quality of life”, and hesitation as to the use of the concept of QoL in the sensitive context of perinatal medicine. It would be interesting to examine in greater depth the significance of this hesitation, to understand that it can be approached either according to principles (deontological approach) or according to consequences (utilitarian approach), neither of which perhaps prevails over the other…

## Competing interests

The authors declare they have no competing interests.

## Author’s contributions

MAE, CG, PLC and PA participated in the conception and design of the study. MAE made the data collection. AL analyzed the data. PA and MAE made data interpretation and participated in the drafting of the article. All the authors contributed to a critical revision of the manuscript and made a substancial contribution to its content. All authors read and approved the final manuscript.

## Pre-publication history

The pre-publication history for this paper can be accessed here:

http://www.biomedcentral.com/1471-2431/13/58/prepub

## Supplementary Material

Additional file 1 Questionnaire: Perceptions, connaissances et attentes des médecins face à l'évaluation de la qualité de vie des enfants nés très grands prématurés. Click here for file
