# Supplementary material for: Quality of life assessment in preterm children: physicians’ knowledge, attitude, belief, practice - a KABP study
Source: BMC Pediatr. 2013 Apr 19;13:58. doi: 10.1186/1471-2431-13-58 (PMC3637183; doi:10.1186/1471-2431-13-58)
Supplement: Additional file 1 — Questionnaire: Perceptions, connaissances et attentes des médecins face à l'évaluation de la qualité de vie des enfants nés très grands prématurés. [file 1471-2431-13-58-S1.doc]

**Questionnaire : Perceptions, connaissances et attentes des médecins face à l'évaluation de la qualité de vie des enfants nés très grands prématurés**

**1. Comment définiriez-vous le concept de Qualité de Vie (QV)?**

**2. Quelles sont vos connaissances du concept de QV ?**

**2.1.** Je connais ce concept car il est devenu une notion fréquemment utilisée dans ma pratique : Oui ❑ Non ❑

**2.2.** J’ai déjà lu des travaux de recherche sur le sujet : Oui ❑ Non ❑

**2.3.** Je connais des questionnaires de QV : Oui ❑ Non ❑

Si oui, vous pouvez préciser votre réponse par des exemples :

**3. Parmi les domaines les plus souvent explorés dans les évaluations de QV destinées aux enfants et aux adolescents, lesquels vous semblent les plus importants ?**

Pour chacun d'entre eux, jugez de leur pertinence (cochez oui ou non) puis, classez en fonction de leur importance ceux pour lesquels vous avez répondu « oui »

(1= le plus important ; 12 = le moins important)

| **Domaines de QV** | **Pertinence** | **Classement** |
| --- | --- | --- |
| 1. Bien-être physique 2. Activités Physiques 3. Symptômes physiques 4. Maladies / Handicap | Oui ❑ Non ❑  Oui ❑ Non ❑  Oui ❑ Non ❑  Oui ❑ Non ❑ | ___  ___  ___  ___ |
| 1. Bien-être Psychologique 2. Image de Soi 3. Émotions et Humeur 4. Comportement Général 5. Fonctionnement cognitif | Oui ❑ Non ❑  Oui ❑ Non ❑  Oui ❑ Non ❑  Oui ❑ Non ❑  Oui ❑ Non ❑ | ___  ___  ___  ___  ___ |
| 1. Relations amicales 2. Relations familiales 3. Vie scolaire | Oui ❑ Non ❑  Oui ❑ Non ❑  Oui ❑ Non ❑ | ___  ___  ___ |

**4. Selon vous, serait-il nécessaire d'ajouter d'autres domaines pour évaluer la QV des enfants nés très grands prématurés (à partir de 6 ans) ?**

Oui ❑ Non ❑ Précisez votre réponse si vous le souhaitez 

**5. A votre avis, les domaines à évaluer doivent-ils être les mêmes, quelque soit l'âge de l'enfant entre 6 et 10 ans ?**

Oui ❑ Non ❑

Si vous le souhaitez, précisez votre réponse

**6. Quel impact attendez-vous d’une étude qui évaluerait la QV des enfants nés très grands prématurés, entre 6 et 10 ans ?**

Pour chacune des affirmations suivantes, veuillez cocher la case de votre choix :

| **6.1. D’un point de vue sociétal, j'attends de cette étude qu'elle...** | Tout à fait d'accord | Plutôt d’accord | Pas tout à fait d’accord | Pas du tout d’accord |
| --- | --- | --- | --- | --- |
| 6.1.1. Apporte une information sur le devenir et la QV de ces enfants au niveau national  6.1.2. Permette que la très grande prématurité devienne une préoccupation de société | ❑  ❑ | ❑  ❑ | ❑  ❑ | ❑  ❑ |

**6.2. Avez-vous d’autres attentes d’un point de vue sociétal ?** Oui ❑ Non ❑ Précisez si vous le souhaitez

| **6.3.** **Concernant l’entourage familial, j'attends de cette étude qu'elle...** | Tout à fait d'accord | Plutôt d’accord | Pas tout à fait d’accord | Pas du tout d’accord |
| --- | --- | --- | --- | --- |
| 6.3.1. Change le regard des parents sur le devenir de leur enfant  6.3.2. Ait un effet sur l'accompagnement familial | ❑  ❑ | ❑  ❑ | ❑  ❑ | ❑  ❑ |

**6. 4. Avez-vous d’autres attentes pour l’entourage familial ?** Oui ❑ Non ❑ Précisez si vous le souhaitez

| **6.5. D’un point de vue médical, disposer de données épidémiologiques concernant la QV …** | Tout à fait d'accord | Plutôt d’accord | Pas tout à fait d’accord | Pas du tout d’accord |
| --- | --- | --- | --- | --- |
| 6.5.1. Permettrait d’avoir une connaissance globale du devenir des patients (retentissement des problèmes physiques, psychiques, sociaux, familiaux)  6.5.2. Permettrait d’informer plus précisément les parents sur le devenir de leur enfant  6.5.3. Permettrait d'insérer le concept de QV dans les pratiques de soins  6.5.4. Améliorerait la communication entre les soignants, le patient et sa famille  6.5.5. Permettrait une analyse et d’éventuelles modifications des pratiques de soins en fonction des résultats obtenus  6.5.6. Ré-engagerait le débat éthique sur les pratiques autour de la réanimation néonatale  6.5.7. N’apporterait rien à ma pratique | ❑  ❑  ❑  ❑  ❑  ❑  ❑ | ❑  ❑  ❑  ❑  ❑  ❑  ❑ | ❑  ❑  ❑  ❑  ❑  ❑  ❑ | ❑  ❑  ❑  ❑  ❑  ❑  ❑ |

**6. 6. Autres attentes d’un point de vue médical ?** Oui ❑ Non ❑ Commentez vos réponses si vous le souhaitez

**6.7. A quel moment ces données concernant la mesure de la QV peuvent être utiles pour les professionnels ?**

- en période périnatale ? Oui ❑ Non ❑

# - dans la pratique à moyen et long terme ? Oui ❑ Non ❑

- autre réponse ? Oui ❑ Non ❑

Veuillez préciser

**7. Dans votre pratique quotidienne, évaluez-vous la QV de vos patients ?** Oui ❑ Non ❑

**7.1. Si oui, précisez la proportion de vos patients bénéficiant de cette évaluation:**

0-25% ❑ 25**-**50% ❑ 50-75% ❑ > 75% ❑

**7.2. Si oui, comment évaluez-vous le plus souvent cette QV ?**

- Au moyen d’un questionnaire standardisé? ❑
- De façon subjective, sans outil particulier ? ❑
- Autre moyen? ❑

Veuillez précisez

Quels seraient les principaux obstacles à l’évaluation de la QV ?

| **8. Je pense que mesurer la QV au moyen d’un questionnaire standardisé :** | Toujours | Souvent | Parfois | Jamais |
| --- | --- | --- | --- | --- |
| 8.1. Enrichirait l'évaluation que je fais intuitivement du bien-être des patients | ❑ | ❑ | ❑ | ❑ |
| 8.2. Donnerait au patient la sensation qu’il est lui-même, plutôt que sa maladie, au centre des préoccupations du médecin  8.3. Faciliterait l'émergence de nouvelles demandes de la part des patients et guiderait la prise en charge des soignants | ❑  ❑ | ❑  ❑ | ❑  ❑ | ❑  ❑ |
| 8.4. N'est pas facile à traiter dans la pratique quotidienne, par manque de temps  8.5. Devrait être confié à d'autres professionnels de santé (psychologues, assistante sociale...) | ❑  ❑ | ❑  ❑ | ❑  ❑ | ❑  ❑ |

**9. Concernant un questionnaire de QV utilisable dans la pratique quotidienne auprès des enfants nés très grands prématurés (entre 6 et 10 ans), quel moyen d'investigation vous semble le plus pertinent? (classez les de 1 à 6 ; 1= le plus pertinent, 6= le moins pertinent)**

L'enfant répond au questionnaire seul I__I

Un parent répond pour l’enfant I__I

Les parents aident l'enfant à répondre au questionnaire I__I

Le médecin référent questionne l'enfant pendant sa consultation I__I

Un soignant non impliqué dans la prise en charge habituelle de l'enfant, aide l'enfant à répondre au questionnaire I__I

Autre proposition I__I Précisez

**9.1. Quel devrait être le nombre maximum de questions d’un tel questionnaire?**

5-10 ❑ 10-15 ❑ 16-20 ❑ > 20 ❑

**9.2. Quel devrait être le temps maximal nécessaire pour remplir un tel questionnaire?**

5 minutes ❑ 10 minutes ❑ 15 minutes ❑ > 15 minutes ❑

**9.3. Quelle modalité de réponse vous paraitrait la plus appropriée ?**

Échelle Visuelle Analogique ❑ Réponse type oui/non ❑ Réponses multiples ❑ Réponse libre ❑

| **9.4. Selon vous, est-il important que le questionnaire de QV...** | Très  important | Assez important | Peu  important | Pas important |
| --- | --- | --- | --- | --- |
| 9.4.1. Donne un score de QV pour chacune des dimensions explorées ?  9.4.2. Donne un score global de QV ?  9.4.3. Soit personnalisé: chaque patient sélectionnant les domaines les plus importants pour lui ?  9.4.4. Soit différent en fonction du handicap de l’enfant ?  9.4.5. Soit le même pour tous les enfants nés très grands prématurés afin de permettre la comparaison entre les différents types de handicaps ? | ❑  ❑  ❑  ❑  ❑ | ❑  ❑  ❑  ❑  ❑ | ❑  ❑  ❑  ❑  ❑ | ❑  ❑  ❑  ❑  ❑ |

**10. Avez-vous d’autres informations ou commentaires dont vous souhaiteriez nous faire part sur la mesure de QV de l’enfant nés très grands prématurés ?**

**Questionnaire supplémentaire : Impact de la mesure de la qualité de vie sur l’éthique de la décision**

**La notion de « qualité de vie » suscite spontanément en vous :**

- de l’intérêt  Oui ❑ Non ❑

- de la perplexité  Oui ❑ Non ❑

**Aborder la QV d’un point de vue éthique**, c’est :

- faire preuve de bienfaisance et d’humanité Oui ❑ Non ❑

- s’interroger sur les possibilités d’autonomie de l’enfant dans l’avenir Oui ❑ Non ❑

- une question de déontologie et de responsabilité professionnelle Oui ❑ Non ❑

**L’appréciation de la qualité de vie :**

- ne peut se faire que de façon subjective et intuitive  Oui ❑ Non ❑

- peut se mesurer de façon plus objective, au moyen de questionnaires  Oui ❑ Non ❑

**Si des données épidémiologiques révélaient une QV très péjorative chez les enfants nés très grands prématurés, cette information**

- aurait une incidence dans vos choix Oui ❑ Non ❑

- demanderait à être relativisée  Oui ❑ Non ❑

- devrait être transmise aux parents au cours de la démarche d’information Oui ❑ Non ❑

**Un renoncement thérapeutique au vu d’une QV future compromise par une affection grave et incurable est-il concevable, selon vous ?** Oui ❑ Non ❑

**Pensez-vous que l’information actuelle concernant les évaluations de la qualité de vie est :**

- Suffisante

- Plutôt insuffisante

- Insuffisante
